# Association between cerebral small vessel disease and plasma levels of LDL cholesterol and homocysteine: Implications for cognitive function

**DOI:** 10.5937/jomb0-50100

**Published:** 2024-09-06

**Authors:** Yan Cheng, Lichao Li, Yafei Lv, Long Zhang, Wenhua Chen, Gongda Xu

**Affiliations:** 1 Gansu Medical College, Affiliated Hospital, Department of Neurology, Pingliang, China

**Keywords:** low-density lipoprotein cholesterol, homocysteine, cerebral small-vessel disease, cognitive functions, holesterol lipoproteina niske gustine, homocistein, cerebralna bolest malih sudova, kognitivne funkcije

## Abstract

**Background:**

Investigate the correlation between low-density lipoprotein (LDL) cholesterol, homocysteine and cognitive function in patients with cerebral small vessel disease (CSVD).

**Methods:**

240 patients with CSVD confirmed by head MRI in the Department of Neurology from January 2020 to December 2023 were retrospectively included in the study. All the patients had complete blood biochemical examination, and their cognitive function was evaluated by Montreal Cognitive Assessment Scale (MoCA), and after correcting for the factor of years of education, the patients were divided into a group of normal cognition (MoCA 26, 70 patients) and a group of cognitive function (MoCA 26, 70 patients) according to the scores. After correcting for the factor of years of education, the patients were divided into the normal cognitive function group (70 cases with MoCA 26) and the cognitive dysfunction group (170 cases with MoCA <26) according to their scores. The general information of the two groups and the patients' cognitive function characteristics, including visuospatial and executive ability, naming, attention and calculation, language, abstraction, delayed memory, and orientation, were compared, and the independent influences on the occurrence of cognitive dysfunction in patients with CSVD were analyzed by two-category multifactorial logistic regression.

**Results:**

Compared with the group with normal cognitive function, the cognitive dysfunction group had lower years of education and higher homocysteine, and the differences were statistically significant (P < 0.05). Compared with the group with normal cognitive functioning, the cognitive dysfunction group had lower MoCA total scores, lower visuospatial and executive ability, naming, attention and calculation, language, abstraction, delayed memory, and orientation scores, and the differences were statistically significant (P < 0.05). Two-category multifactorial logistic regression analysis showed that low-density lipoprotein cholesterol (OR=2.756, 95% CI: 0.673-0.938, P=0.012) and homocysteine (OR=1.859, 95% CI: 1.024-1.324, P=0.016) were the independent factors influencing cognitive dysfunction in CSVD patients. The lower the risk of cognitive impairment in CSVD patients, the higher the plasma LDL cholesterol and homocysteine levels, the higher the risk of cognitive impairment in CSVD patients.

**Conclusions:**

Plasma LDL cholesterol and homocysteine levels are associated with and may be predictors of cognitive dysfunction in patients with CSVD.

## Abstract (sr)

**Uvod:**

Cilj je bio da se istraži korelacija između holesterola lipoproteina niske gustine (LDL), homocisteina i kognitivne funkcije kod pacijenata sa bolešću malih sudova mozga (CSVD).

**Metode:**

U studiju je retrospektivno uključeno 240 pacijenata sa CSVD potvrđenim glavnim Mr na Odeljenju za neurologiju od januara 2020. do decembra 2023. godine. Svi pacijenti su imali kompletan biohemijski pregled krvi, a njihova kognitivna funkcija je procenjena Montrealskom skalom kognitivne procene (MoCA), a nakon korekcije faktora godina obrazovanja, pacijenti su podeljeni u grupu normalne kognitacije (MoCA 26, 70 pacijenata) i grupa kognitivnih funkcija (MoCA 26, 70 pacijenata) prema rezultatima. Nakon korekcije faktora godina obrazovanja, pacijenti su podeljeni u grupu sa normalnim kognitivnim funkcijama (70 slučajeva sa MoCA 26) i grupu sa kognitivnom disfunkcijom (170 slučajeva sa MoCA <26) prema rezultatima. Upoređene su opšte informacije dve grupe i karakteristike kognitivnih funkcija pacijenata, uključujući vizuelno-prostorne i izvršne sposobnosti, imenovanje, pažnju i računanje, jezik, apstrakciju, odloženo pamćenje i orijentaciju, kao i nezavisni uticaji na pojavu kognitivne disfunkcije. Kod pacijenata sa CSVD analizirane su dve kategorije multifaktorske logističke regresije.

**Rezultati:**

U poređenju sa grupom sa normalnom kognitivnom funkcijom, grupa sa kognitivnom disfunkcijom je imala niže godine obrazovanja i viši nivo homocisteina, a razlike su bile statistički značajne (P < 0,05). U poređenju sa grupom sa normalnim kognitivnim funkcionisanjem, grupa sa kognitivnom disfunkcijom imala je niže ukupne rezultate MoCA, niže vizuelno-prostorne i izvršne sposobnosti, imenovanje, pažnju i računanje, jezik, apstrakciju, odloženo pamćenje i orijentaciju, a razlike su bile statistički značajne (P < 0,05). Dvokategorijska multifaktorska logistička regresiona analiza pokazala je da su holesterol lipoproteina niske gustine (OR=2,756, 95% CI: 0,673-0,938, P=0,012) i homocistein (OR=1,859, 95% CI: 1,024-1,324, P=0,016) bili nezavisni faktori koji utiču na kognitivnu disfunkciju kod pacijenata sa CSVD. Što je manji rizik od kognitivnog oštećenja kod pacijenata sa CSVD, što je viši nivo LDL holesterola i homocisteina u plazmi, veći je rizik od kognitivnog oštećenja kod pacijenata sa CSVD.

**Zaključak:**

Nivoi LDL holesterola i homocisteina u plazmi su povezani i mogu biti prediktori kognitivne disfunkcije kod pacijenata sa CSVD.

## Introduction

As populations age and human life expectancy increases, age-related diseases pose significant challenges to society and health care systems. There are currently more than 40 million people living with dementia worldwide and this is expected to double every 20 years. cerebral small vessel disease (CSVD) is the most important vascular risk factor for dementia, accounting for 36% to 67% of vascular dementia (VaD). It also interacts with Alzheimer’s disease (AD) and other types of dementia, increasing the risk of dementia. Cerebral small vessel disease (CSVD) is predominantly characterized by the involvement of small arteries and veins, encompassing conditions such as small vessel disease of the white matter, microvascular disease, and blood-brain barrier disruption [1] [2]. Not only is CSVD a common etiology of cognitive decline and dementia in the elderly, but it is also closely associated with neurological impairments including stroke, motor disorders, and depression [3] [4] [5].

Low-density lipoprotein cholesterol (LDL-C) and homocysteine (hcy) are two biochemical markers present in the bloodstream, intimately linked to cardiovascular diseases and metabolic syndrome. Recent investigations have suggested a potential relationship between these two markers and the development of CSVD as well as cognitive decline [6]. Elevated LDLC levels primarily contribute to atherosclerosis and vascular inflammation, establishing a significant connection to the pathological processes underlying vascular diseases.

Some studies have reported a certain correlation between hypercholesterolemia and the occurrence of cerebral small vessel disease (CSVD) as well as cognitive decline. Additionally, homocysteine, a sulfur-containing amino acid, is associated with metabolic disruption, oxidative stress, and inflammatory reactions [4]. Existing research has demonstrated a link between elevated homocysteine levels and cerebral vascular damage, along with cognitive decline. However, studies investigating the correlation between plasma low-density lipoprotein cholesterol (LDL-C) and homocysteine levels, and cognitive function in CSVD patients, remain relatively limited. 

Cognitive impairment caused by CSVD typically exhibits an insidious onset and a slow progression, often leading to under-recognition during the early stages. By the time patients are initially diagnosed, neurological damage may be difficult to reverse [7]. further contributing to the burden of the disease. Therefore, the objectives of this study are to identify relevant risk factors, particularly modifiable ones, for cognitive impairment in CSVD and to provide further evidence for the clinical management of cognitive impairment in CSVD patients. This study aims to shed light on the identification of related risk factors for cognitive impairment in CSVD and especially focus on modifiable risk factors, providing additional insights for the clinical management of cognitive impairment in CSVD patients.

## Materials and methods

### Objects

Two hundred and forty patients with CSVD confirmed by head MRI in the Department of Neurology from January 2020 to December 2023 were retrospectively included in the consecutive enrollment. All patients had complete blood biochemistry examination, and cognitive function was evaluated by Montreal Cognitive Assessment Scale (MoCA), and after correcting for the factor of years of schooling, the patients were divided into cognitively normal group (MoCA 26 points, 70 cases) and cognitively impaired group (MoCA <26 points, 170 cases) according to the scoring results. The patients were divided into the normal cognitive function group (70 cases with MoCA 26 points) and the cognitive dysfunction group (170 cases with MoCA <26 points).

Inclusion criteria: (1) Participants must be 18 years of age or older, with no gender restrictions; (2) Participants must meet the diagnostic criteria for CSVD as outlined in the 2021 Chinese Expert Consensus on the Diagnosis and Treatment of Cerebral Small Vessel Disease and must have received confirmation through head MRI imaging.

Exclusion criteria: (1) Patients with alternative causes of stroke such as atherosclerotic cerebral infarction, cardiac cerebral embolism, traumatic brain injury, cerebral hemorrhage, intracranial space-occupying lesions, or other intracranial abnormalities will be excluded; (2) Patients with multiple organ failure, hematological diseases, or advanced systemic malignant tumors will be excluded; (3) Patients unable to undergo or cooperate with necessary study procedures, including head MRI and cognitive function assessments, will be excluded. The study protocol was approved by the hospital’s Ethics Committee.

## Methods

Blood tests (morning blood after at least 8 hours of fasting): Renal function assessment includes creatinine (normal reference range: 44–133 μmol/L), estimated glomerular filtration rate (eGFR; normal reference range: 80–120 mmol/L – 1.73 m^2^), urea (normal reference range: 1.8–1.73 m^2^), and blood glucose (normal reference range: 1.5–1.5 m^2^), reference range: 80–120 mmol/L -1.73 m^2^), urea (normal reference range: 1.8–7.1 mmol/L) and uric acid (normal reference range: 150–420 μmol/L), fasting glucose (normal reference range: 3.61–6.11 mmol/L), and ultrasensitive C-reactive protein (normal reference range: 3.61–6.11 mmol/L).Lipids include total cholesterol (normal reference range: 3.4–5.2 mmol/L), HDL cholesterol (normal reference range: 0.9–1.4 mmol/L), LDL cholesterol (normal reference range: 2.1–3.1 mmol/L), and triacylglycerol (normal reference range: 0.56–1.5 mmol/L). Reference range: 0.56–1.7 mmol/L), homocysteine (normal reference range: 6–17 μmol/L); current statin use.

The diagnostic criteria for hypertension, diabetes mellitus and hyperlipidemia were referred to the Chinese Guidelines for the Prevention and Control of Hypertension (2018 Revision) [8], the Chinese Guidelines for the Prevention and Control of Type 2 Diabetes Mellitus (2020 Revision) [9] and the Chinese Guidelines for the Prevention and Control of Dyslipidemia in Adults (2016 Revision) [10], respectively. Smoking history was defined as continuous smoking for more than 6 months with an average of more than 10 cigarettes/d, and drinking history was defined as drinking for more than 6 months with an average alcohol intake of more than 30 g/d or 210 g/week [11]. The eGFR was calculated according to the Cockcroft-Gault formula [12]: eGFR = (140-age) x body mass (kg)/[0.818 x blood creatinine (μmol/L)] for men and Female Ccr= (140-age) × Body weight (kg) × 1.03/ serum creatinine (μmol/L).

### Cognitive assessment

Cognitive assessment was conducted using the Montreal Cognitive Assessment (MoCA) tool [13], which comprises 7 subcognitive domains: visuospatial and executive ability, naming, attention and calculation, language, abstraction, delayed memory, and orientation. The total MoCA score ranges from 0 to 30. To account for educational differences, an additional point was added to the total score for patients with less than 12 years of education. Patients were categorized into two groups based on cognitive function: a cognitively normal group (MoCA score 26 points) and a cognitively impaired group (MoCA score <26 points). However, the specific criteria used for categorization and the detailed process of cognitive assessment were not clearly defined in the methods section. Including more detailed information on these aspects would improve the methodological rigor and reproducibility of the study.

### Statistical methods

The study employed several statistical methods to investigate the correlation between LDL cholesterol, homocysteine, and cognitive function in patients with cerebral small vessel disease (CSVD). General information and cognitive function characteristics of the two groups were compared using descriptive statistics to provide an overview of the patient population. Statistical tests such as t-tests or nonparametric equivalents were used to compare the differences in years of education, homocysteine levels, and MoCA scores between the group with normal cognitive function and the cognitive dysfunction group. Two-category multifactorial logistic regression analysis was performed to identify independent factors influencing cognitive dysfunction in CSVD patients. This analysis assessed the relationship between LDL cholesterol, homocysteine levels, and the occurrence of cognitive dysfunction while controlling for other potential confounding variables. Odds ratios (OR) and 95% confidence intervals (CI) were reported to quantify the strength of association between LDL cholesterol, homocysteine levels, and cognitive dysfunction. Statistical significance was determined using P-values, with values less than 0.05 considered significant.

## Results

### General information

Compared with the patients in the group with normal cognitive function, the patients in the cognitive dysfunction group had lower years of education and higher levels of low-density lipoprotein cholesterol and homocysteine, and the differences were statistically significant (P < 0.05). See Table 1.

**Table 1:** Comparison of general data of patients. Note: BMI is body mass index, eGFR is estimated glomerular filtration rate; ^a^is t value and ^b^is 2 is the value, and ^c^is the z value

	Cognitively normal group (n=70)	Cognitive dysfunction group (n=170)	χ^2^/F value	P value
Age (x̄±s, year)	65.2±14.4	66.8±11.3	-1.784^a^	0.156
Masculinity [n(%)]	50(71.4)	104(61.2)	1.679^b^	0.304
Education years [M(P25,P75), year]	13(12, 17)	10(8,12)	-3.167^e^	0.042
BMI (x̄±s, year)	26.7±4.26	27.1±4.79	1.673^a^	0.278
Hypertension [n(%)]	60(85.7)	122(71.8)	3.157^b^	0.127
Diabetes [n(%)]	24(34.3)	44(25.9)	0.486^b^	0.158
Hyperlipemia [n(%)]	54(77.1)	122(71.8)	0.782^b^	0.356
Statins [n(%)]	52(74.3)	112(65.9)	0.892^b^	0.429
Smoking history [n(%)]	44(62.9)	82(48.2)	2.178^b^	0.126
Systolic pressure (x̄±s, mmHg)	134.7±17.2	142.6±22.6	-1.782^a^	0.172
Diastolic pressure (x̄±s, mmHg)	82.7±16.8	81.6±9.2	0.562^a^	0.479
Heart rate (x̄±s/min)	75.3±10.6	75.9±11.2	0.765^a^	0.705
Creatinine [M(P25,P75), μmol/L]	78.0(67.9, 92.6)	79.2(68.5, 91.7)	-0.502^e^	0.809
Egfr [M(P25,P75]	85.06(70.67, 94.27)	82.78(75.02, 90.89)	-0.506^e^	0.708
Urea [M(P25,P75), mmol/L]	5.46(4.56, 6.74)	5.28(4.37, 6.83)	-0.573^e^	0.673
UA [(x̄±s, μmol/L)]	345.7±67.89	309±89.56	-0.783^a^	0.302
FBG [(x̄±s, mmol/L)]	6.26±1.67	5.78±1.89	-0.908^a^	0.152
hs-CRP [M(P25,P75), mg/L]	1.56(0.67, 2.18)	1.07(0.63, 2.37)	-0.987^e^	0.275
TCHO [(x̄±s, μmol/L)]	3.87±1.26	4.04±1.56	-1.562^a^	0.106
TG [M(P25, P75)]	1.16(0.83, 1.95)	1.19(0.92, 1.89)	-0.705^e^	0.605
HDL-C [M(P25, P75)]	1.06(0.83, 1.25)	1.08(0.87, 1.45)	-2.062^e^	0.221
LDL-C [(x̄±s)]	2.07±0.45	2.25±0.38	-2.806^a^	0.012
Hcy [M( P25, P75), μmol/L]	12.78(9.67, 18.27)	1.56(11.6, 18.56)	-2.408^e^	0.023

### Comparison of cognitive function between the two groups of patients

Compared with the group with normal cognitive function, the cognitive dysfunction group had a lower MoCA total score and its various sub-cognitive domain scores, and the differences were statistically significant (all P < 0.05). See Table 2.

**Table 2:** Comparison of cognitive function in patients with small cerebral vascular disease. Note: MoCA is the Montreal Cognitive Assessment Scale

	Cognitively normal group (n=70)	Cognitive dysfunction group (n=170)	Z value	*P* value
MoCA score [M(P25, P75), score]	27.0(26.0,29.0)	21.0(17.0,24.0)	-9.792	0.001
Visual space and executive ability [M(P25, P75), score]	5.0(4.0,5.0)	3.0(2.0,4.0)	-7.351	0.002
Namespace [M(P25, P75), score]	3.0(3.0,3.0)	3.0(2.0,3.0)	-3.026	0.012
Attention and numeracy [M(P25, P75), score]	6.0(6.0,6.0)	6.0(4.0,6.0)	-4.056	0.025
Language [M(P25, P75), score]	3.0(2.0,3.0)	2.0(1.0,2.0)	-4.682	0.017
Abstraction [M(P25, P75), score]	2.0(2.0,2.0)	1.0(1.0,2.0)	-5.726	0.019
Delayed Memory [M(P25, P75), score]	4.0(3.0,5.0)	1.0(0.0,2.0)	-2.783	0.025
Orientation [M(P25, P75), score]	6.0(6.0,6.0)	5.0(5.0,6.0)	-2.056	0.021

### Dichotomous multifactorial logistic regression analysis

Binary Logistic regression analysis showed that years of education, low density lipoprotein cholesterol and homocysteine were independent factors affecting cognitive dysfunction in CSVD patients. The higher the levels of low-density lipoprotein cholesterol and homocysteine, the higher the risk of cognitive impairment in CSVD patients; The higher the number of years of education, the lower the risk of cognitive impairment in CSVD patients. See Table 3.

**Table 3:** Multivariate Logistic regression analysis of cognitive dysfunction. Note: LDL-C is low-density lipoprotein cholesterol; HCY is Homocysteine.

Factor	value	*SE*	Wald χ^2^	P value	*OR* value	95%*CI*
LDL-C	0.879	0.372	5.891	0.012	2.756	0.673–0.938
HCY	0.125	0.057	4.268	0.016	1.859	1.024–1.324

## Discussion

Cerebral small-vessel disease refers to diseases with clinical, cognitive, imaging and pathological manifestations caused by small-vessel lesions in the brain. Studies in China have shown that lacunar cerebral infarction accounts for 42.3% of all ischemic stroke causes [14]. Among them, cognitive decline is the most common and important clinical manifestation of cerebral small vessel disease. Therefore, early identification and intervention at the VCIND or VaMCI stage is a key target for the prevention and treatment of VaD and is of great clinical significance. Currently, it is believed that CSVD is caused by various factors, and lipid metabolism disorder is one of the risk factors for CSVD, and among the lipid metabolism disorders, the increase of LDL level is the most harmful one, and CSVD can be prevented by controlling the level of LDL in clinical practice [15]. The pathology of cerebral small vessel disease is characterized by atherosclerosis of small arteries, i.e., loss of smooth muscle in the vessel wall, diffuse lipid deposition, infiltration of plasma proteins and inflammatory factors, and formation of lipid hyalinosis, and LDL-C, the main component of cholesterol, is an important factor in the formation of atherosclerosis.

Cerebral small vessel disease is a common risk factor for stroke and a major cause of vascular cognitive impairment. Cerebral microangiopathy and neurodegeneration are closely associated with cognitive decline and dementia in the elderly [16]. Relevant studies have found that CSVD is a dynamic disease, and the degree of cognitive decline caused by cerebral microangiopathy depends on the progression of microangiopathy [17]. VHyperhomocysteinemia (HHcy) is also recognized as a risk factor for Vascular cognitive impairment (VCI). Wang et al. [18] found that Hcy levels are strongly associated with cerebral small vessel disease and are considered an independent risk factor for CSVD. Hcy is an endothelial toxin that causes vascular injury by promoting oxidative damage in arteries, destroying vascular matrix, and increasing the proliferation of vascular smooth muscle cells, and also alters blood coagulation properties and disrupts endothelium-dependent diastolic regulation of the vasculature, which further causes cognitive dysfunction [19].

The present study focused on exploring the association of Hcy levels and LDL-C with cognitive impairment in CSVD. Hcy has received widespread attention as a risk factor for cognitive impairment [20]. The results of this study showed that Hcy and LDL is an independent risk factor for cognitive dysfunction in CSVD, and age, gender, smoking history, drinking history, hypertension, diabetes mellitus, uric acid, fasting glucose, triglycerides, total cholesterol, and low-density lipoprotein (LDL-C), which were mentioned above, did not affect the level of Hcy. Recent studies have demonstrated that Hcy acts as a neurotoxin that promotes neurodegeneration through apoptosis caused by DNA breaks [21]. HHcy has been reported to be a risk factor for VCI in patients with cerebral infarction and an independent risk factor for MCI in the Xinjiang Uyghur population [22] [23]. Wang et al. [16] demonstrated that serum Hcy levels correlated with the occurrence of vascular MCI , and that cognitive impairment may be caused by increased cerebrovascular disease-associated cortical or hippocampal atrophy as a result of the toxic injury of a high Hcy In addition, serum Hcy levels were positively associated with vascular MCI in patients with CSVD and may serve as a predictor of vascular MCI [16]. Feng et al. [24] demonstrated that Hcy is more closely associated with small vessel disease than large vessel disease. Jakubowski et al. et al. [25] demonstrated that Hcy in general is an independent risk factor for CSVD, and that Hcy toxic effects may include direct endothelial injury or triggering endothelial inflammation. The toxic effects of Hcy may include direct endothelial damage or triggering endothelial inflammatory responses. The results of the present study are consistent with these studies and suggest that elevated levels of Hcy lead to cognitive impairment in CSVD, and that the pathogenesis of CSVD is based on the following mechanisms: 1) Hcy is endotoxic and neurotoxic to the vascular endothelium, and 2) Hcy has a post-translational modification of proteins known as homocysteinylation, which is toxic to the nerves [25] [26].

Meanwhile, the results of this study showed that LDL cholesterol levels were higher in the cognitive dysfunction group than in the cognitive function group, and logistic regression analysis showed that LDL cholesterol was an independent risk factor for cognitive dysfunction in patients with CSVD, suggesting that LDL cholesterol may be involved in the development of cognitive dysfunction in CSVD. Todate et al. [8] showed that the prevalence of periventricular white matter high signal was significantly higher than that of healthy controls in patients with familial hypercholesterolemia. Todate et al. [8] showed that the prevalence of periventricular white matter hyperintensities in patients with familial hypercholesterolemia was significantly higher than that in healthy controls, and the prevalence of deep white matter hyperintensities was also on the rise in familial hypercholesterolemia. Imamura et al. [27] followed up 2,351 residents aged 40 years of a certain community for 19 years, and found that the patients with high levels of LDL-cholesterol had a higher risk of cavernous cerebral defects. It was found that patients with high levels of LDL cholesterol had a higher incidence of lacunar cerebral infarction. According to national and international guidelines, CSVD may cause cognitive decline [3] [28]. The present study also supports that high levels of LDL cholesterol are associated with cognitive impairment in CSVD.

There is a positive association between LDL-C and small cerebral vessel disease: high levels of LDLC may be an important risk factor for small cerebral vessel disease. Long-term high levels of LDL-C can lead to vascular endothelial damage, atherosclerosis and small brain vessel damage, thereby increasing the risk of small brain vessel disease. There is a positive association between homocysteine and small cerebral vessel diseases: high levels of homocysteine are closely related to the occurrence and development of small cerebral vessel diseases. Accumulation of homocysteine can cause endothelial cell damage, inflammatory responses, and changes in blood vessel walls, thereby increasing the risk of brain small vessel disease. High levels of LDL-C and homocysteine are associated with cognitive decline: Brain small vessel disease is strongly associated with cognitive decline. High levels of LDL-C and homocysteine may further impair brain function by promoting mechanisms such as cerebrovascular injury, ischemia, and inflammatory response, leading to cognitive decline. Based on the above conclusions, rational control of plasma LDL-C and homocysteine levels may help reduce the risk of brain small vessel disease and may improve cognitive function. LDL-C and homocysteine levels can be effectively controlled through appropriate lifestyle interventions, such as rational diet, active exercise and drug therapy, thereby preventing the development of brain small vessel disease and improving cognitive performance.

In this study, there are several limitations that should be acknowledged. Firstly, the retrospective design of the study may introduce biases and limit the ability to establish causal relationships between lowdensity lipoprotein (LDL) cholesterol, homocysteine, and cognitive function in patients with cerebral small vessel disease (CSVD). Additionally, the sample size of 240 patients from a single department may not be representative of the broader population with CSVD, potentially affecting the generalizability of the findings. Furthermore, the use of the Montreal Cognitive Assessment Scale (MoCA) for evaluating cognitive function, while widely used, may not capture the full spectrum of cognitive abilities, potentially overlooking certain aspects of cognitive impairment in patients with CSVD. The study also lacks detailed information on other potential confounding factors, such as lifestyle habits, comorbidities, or medication use, which could influence the relationship between LDL cholesterol, homocysteine, and cognitive function. Moreover, the study did not address the longitudinal changes in LDL cholesterol and homocysteine levels over time or their potential impact on cognitive function in CSVD patients. Therefore, further prospective investigations are needed to better understand the causal mechanisms and long-term implications of these biomarkers on cognitive impairment in CSVD.

In conclusion, MoCA examination of CSVD patients for early identification of cognitive dysfunction, detection of plasma LDL cholesterol and homocysteine levels in CSVD patients, and timely diagnosis and treatment may have a predictive effect on the occurrence and development of cognitive dysfunction in CSVD patients. However, the shortcoming of this study is that it is a single-center, small-sample retrospective analysis, and the degree of cognitive dysfunction of CSVD patients was not graded, which needs to be further explored and verified by subsequent multi-center, large-sample, and more detailed analysis data.

## Dodatak

### Ethical compliance 

This study was approved by the ethics committee of Affiliated Hospital of Gansu Medical College. 

### Author contributions

YC and GX designed the study and performed the experiments, LL and YL collected the data, LZ and WC analyzed the data, YC and GX prepared the manuscript. All authors read and approved the final manuscript.

Yan Cheng and Lichao Li contributed equally to this work.

### Funding

This study did not receive any funding in any form.

### Conflict of interest statement

All the authors declare that they have no conflict of interest in this work.

## References

[1] Kim J S (2021). Role of Blood Lipid Levels and Lipid-Lowering Therapy in Stroke Patients with Different Levels of Cerebral Artery Diseases: Reconsidering Recent Stroke Guidelines. J Stroke.

[2] Liu Y, Yuan C, Chen X, Fang X, Hao J, Zhou M, et al (2023). Association of Plasma Lipids with White Matter Hyperintensities in Patients with Acute Ischemic Stroke. Int J Gen Med.

[3] van Nieuwkerk A C, Delewi R, Wolters F J, Muller M, Daemen M, Biessels G J (2023). Cognitive Impairment in Patients With Cardiac Disease: Implications for Clinical Practice. Stroke.

[4] Chen Y, Hu M, Gong H (2017). Correlation analysis between the LDL-C in serum and the onset of transient ischemic attack caused by CSVD. Exp Ther Med.

[5] Li C, Bu X, Liu Y (2021). Effect of folic acid combined with pravastatin on arteriosclerosis in elderly hypertensive patients with lacunar infarction. Medicine.

[6] Amarenco P, Benavente O, Goldstein L B, Callahan A R, Sillesen H, Hennerici M G, et al (2009). Results of the Stroke Prevention by Aggressive Reduction in Cholesterol Levels (SPARCL) trial by stroke subtypes. Stroke.

[7] Beltran R L, Vallejo-Vaz A J, Muniz G O (2021). Cerebrovascular Disease and Statins. Front Cardiovasc Med.

[8] Todate Y, Uwano I, Yashiro S, Chida A, Hasegawa Y, Oda T, et al (2019). High Prevalence of Cerebral Small Vessel Disease on 7T Magnetic Resonance Imaging in Familial Hypercholesterolemia. J Atheroscler Thromb.

[9] Georgakis M K, Malik R, Anderson C D, Parhofer K G, Hopewell J C, Dichgans M (2020). Genetic determinants of blood lipids and cerebral small vessel disease: Role of high-density lipoprotein cholesterol. Brain.

[10] Zhang J, Liu N, Yang C (2019). Effects of rosuvastatin in combination with nimodipine in patients with mild cognitive impairment caused by cerebral small vessel disease. Panminerva Med.

[11] Zhu S, Wei X, Yang X, Huang Z, Chang Z, Xie F, et al (2019). Plasma Lipoprotein-associated Phospholipase A2 and Superoxide Dismutase are Independent Predicators of Cognitive Impairment in Cerebral Small Vessel Disease Patients: Diagnosis and Assessment. Aging Dis.

[12] Xin J, Huang X, Pan X, Lin L, Sun M, Liu C, et al (2019). Risk Factors for Aphasia in Cerebral Small Vessel Diseases. Curr Neurovasc Res.

[13] Tang H, Wang Y, Cheng A, Wang A, Xu J, Zhang C, et al (2020). Association between Low-Density Lipoprotein Cholesterol Levels and Proximal Single Subcortical Infarction in Comparison with Distal Single Subcortical Infarction. J Stroke Cerebrovasc Dis.

[14] Zhao Y, Zhou Y, Zhou H, Gong X, Luo Z, Li J, et al (2023). Low-density lipoprotein cholesterol, statin therapy, and cerebral microbleeds: The CIRCLE study. Neuroimage Clin.

[15] Kang S H, Yoo H, Cheon B K, Park Y H, Kim S J, Ham H, et al (2023). Distinct effects of cholesterol profile components on amyloid and vascular burdens. Alzheimers Res Ther.

[16] Wang R, Laveskog A, Laukka E J, Kalpouzos G, Backman L, Fratiglioni L, et al (2018). MRI load of cerebral microvascular lesions and neurodegeneration, cognitive decline, and dementia. Neurology.

[17] Hilal S, Mok V, Youn Y C, Wong A, Ikram M K, Chen C L (2017). Prevalence, risk factors and consequences of cerebral small vessel diseases: data from three Asian countries. J Neurol Neurosur Ps.

[18] Wang T, Sun Z W, Shao L Q, Xu X B, Liu Y, Qin M, et al (2017). Diagnostic Values of Serum Levels of Homocysteine and Uric Acid for Predicting Vascular Mild Cognitive Impairment in Patients with Cerebral Small Vessel Disease. Med Sci Monitor.

[19] Wang C Y, Chen Z W, Zhang T, Liu J, Chen S H, Liu S Y, et al (2014). Elevated plasma homocysteine level is associated with ischemic stroke in Chinese hypertensive patients. Eur J Intern Med.

[20] Lehmann M, Gottfries C G, Regland B (1999). Identification of cognitive impairment in the elderly: Homocysteine is an early marker. Dement Geriatr Cogn.

[21] Sachdev P S (2005). Homocysteine and brain atrophy. Prog Neuro-Psychoph.

[22] An X L, Li C L (2015). Analysis of risk factors for vascular cognitive impairment in patients with cerebral infarction. Cell Biochem Biophys.

[23] Luo M, Ji H, Zhou X, Liang J, Zou T (2015). Correlation of homocysteine metabolic enzymes gene polymorphism and mild cognitive impairment in the Xinjiang Uygur population. Med Sci Monitor.

[24] Feng C, Bai X, Xu Y, Hua T, Huang J, Liu X Y (2013). Hyperhomocysteinemia associates with small vessel disease more closely than large vessel disease. Int J Med Sci.

[25] Jakubowski H (1999). Protein homocysteinylation: possible mechanism underlying pathological consequences of elevated homocysteine levels. Faseb J.

[26] Perla-Kajan J, Twardowski T, Jakubowski H (2007). Mechanisms of homocysteine toxicity in humans. Amino Acids.

[27] Imamura T, Doi Y, Arima H, Yonemoto K, Hata J, Kubo M, et al (2009). LDL cholesterol and the development of stroke subtypes and coronary heart disease in a general Japanese population: the Hisayama study. Stroke.

[28] Skrobot O A, Black S E, Chen C, Decarli C, Erkinjuntti T, Ford G A, et al (2018). Progress toward standardized diagnosis of vascular cognitive impairment: Guidelines from the Vascular Impairment of Cognition Classification Consensus Study. Alzheimers Dement.

